# Distribution and reference interval establishment of neutral‐to‐lymphocyte ratio (NLR), lymphocyte‐to‐monocyte ratio (LMR), and platelet‐to‐lymphocyte ratio (PLR) in Chinese healthy adults

**DOI:** 10.1002/jcla.23935

**Published:** 2021-08-13

**Authors:** Junjun Wang, Fan Zhang, Feng Jiang, Lijuan Hu, Jian Chen, Yumin Wang

**Affiliations:** ^1^ Department of Laboratory Medicine the First Affiliated Hospital of Wenzhou Medical University Wenzhou China

**Keywords:** lymphocyte‐to‐monocyte ratio, neutrophil‐to‐lymphocyte ratio, platelet‐to‐lymphocyte ratio, reference interval

## Abstract

**Background:**

Neutral‐to‐lymphocyte ratio (NLR), lymphocyte‐to‐monocyte ratio (LMR), and platelet‐to‐lymphocyte ratio (PLR) are associated with coronavirus disease 2019 (COVID‐19) and many diseases, but there are few data about the reference interval (RI) of NLR, LMR, and PLR.

**Methods:**

The neutrophil count, lymphocyte count, monocyte count, and platelet count of 404,272 Chinese healthy adults (>18 years old) were measured by Sysmex XE‐2100 automatic hematology analyzer, and NLR, LMR, and PLR were calculated. According to CLSI C28‐A3, the nonparametric 95% percentile interval is defined as the reference interval.

**Results:**

The results of Mann‐Whitney U test showed that NLR (*p* < .001) in male was significantly higher than that in female; LMR (*p* < .001) and PLR (*p* < .001) in male were significantly lower than that in female. Kruskal‐Wallis H test showed that there were significant differences in NLR, LMR, and PLR among different genders and age groups (*p* < .001). The linear graph showed that the reference upper limit of NLR and PLR increased with age and the reference upper limit of LMR decreases with age in male population. In female population, the reference upper limit of NLR in 50–59 group, LMR in >80 group, and PLR in 70–79 group appeared a trough; the reference upper limit of NLR in >80 group, LMR in 50–59 group, and PLR in 40–49 group appeared peak.

**Conclusion:**

The establishment of RI for NLR, LMR, and PLR in Chinese healthy adults according to gender and age will promote the standardization of clinical application.

## Graphical abstract

Values of upper reference limits for three indicators in different age partitions. A: Values of upper reference limits for NLR in different age partitions. B: Values of upper reference limits for LMR in different age partitions. C: Values of upper reference limits for PLR in different age partitions. The line chart based on age showed that the reference upper limit of NLR, LMR, and PLR increased with age in male population. In female population, the reference upper limit of NLR in 50–59 group, LMR in >80 group, and PLR in 70–79 group showed a trough; the reference upper limit of NLR in >80 group, LMR in 50–59 group, and PLR in 40–49 group showed peak.

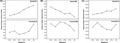

## 1 INTRODUCTION

With the deepening of the research on inflammatory markers, there is a growing interest in research aimed at better understanding the disease status or predicting the prognosis of patients with simple blood inflammatory markers. Neutrophil‐to‐lymphocyte ratio (NLR), lymphocyte‐to‐monocyte ratio (LMR), and platelet‐to‐lymphocyte ratio (PLR) are novel biomarkers of systemic inflammation, which are closely related to immune response.

Studies have shown that NLR, LMR, and PLR are a independent risk factor for coronavirus disease 2019 (COVID‐19)^1^, ^2^, ^3^ and can predictive of response to chemotherapy and survival in patients with malignancy.^4^, ^5^, ^6^, ^7^, ^8^ They are also associated with a variety of other diseases. Platelet‐to‐lymphocyte ratio (PLR) has been suggested to be associated with type 2 diabetes mellitus^9^ and bowel disease.^10^ PLR can also be used as a prognostic marker of fibrosis in patients with NAFLD and can be disturbed in patients with liver cirrhosis.^11^ On the other hand, neutrophil‐to‐lymphocyte ratio (NLR) has been suggested to be associated with cardiac arrhytmia,^12^ inflammatory bowel disease,^13^ thyroiditis,^14^ malignant nodules,^15^ diabetes mellitus,^16^ and irritable bowel syndrome.^17^ Similarly, lymphocyte‐to‐monocyte ratio (LMR) or reversely monocyte‐to‐lymphocyte ratio has been related to diabetic foot,^18^ breast cancer,^19^ and diabetic kidney injury.^20^

Therefore, these markers have been widely used in clinical practice. However, due to the influence of genotype, geographical location, lifestyle, and many other factors, these markers may be different among different regions, genders, and ages. Therefore, we urgently need to establish a specific and reliable reference interval (RI) for NLR, LMR, and PLR in Chinese population.

Prior to that, some scholars have carried out relevant research on the reference intervals of NLR, LMR, and PLR.^21^, ^22^, ^23^ However, the results are not very representative due to the small amount of data and incomplete coverage area. In this study, 404,272 healthy adults were tested and the reference intervals of NLR, LMR, and PLR were established according to Clinical and Laboratory Standards Institute (CLSI) CA28‐A3.^24^

## 2 MATERIAL AND METHODS

### 2.1 Study subjects

**FIGURE 1:**
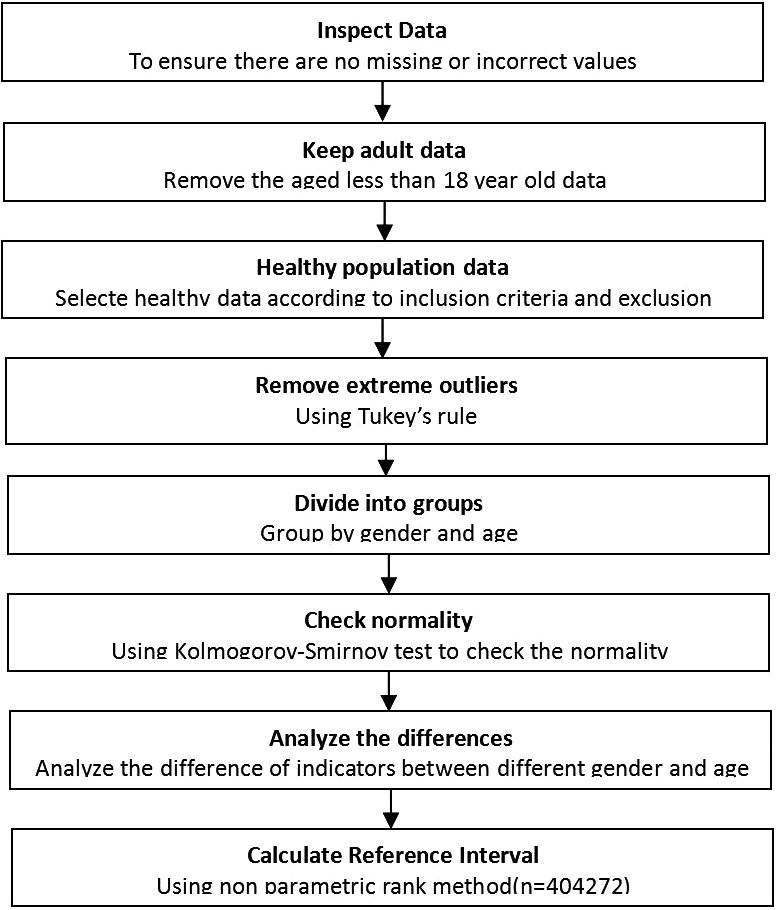
Establishing reference interval of neutrophil‐to‐lymphocyte ratio, lymphocyte‐to‐monocyte ratio, and platelet‐to‐lymphocyte ratio on the bias of Clinical and Laboratory Standards Institute CA28‐A3

According to the inclusion and exclusion criteria, 404,272 healthy adults (205,592 men, 18–112 years old; 198,680 women, 18–112 years old) who completed physical examination in the physical examination center of the First Affiliated Hospital of Wenzhou Medical University from January 2009 to December 2019 were selected as the research objects. According to CLSI C28‐A3, the subjects were divided into 12 groups according to gender (male and female) and age (18–39 years old; 40–49 years old; 50–59 years old; 60–69 years old; 70–79 years old; and >80 years old) to determine whether the distribution of NLR, LMR, and PLR varied with age and / or gender. The detailed screening procedures of the study participants are shown in Figure 1.

Inclusion criteria were as follows: (a) adults over 18 years old; (b) no history of surgery in the past 6 months; (c) no leukemia or any other blood system diseases; (d) no receiving transfusion of blood or any other blood products; (e) infectious diseases such as hepatitis B virus, hepatitis C virus, syphilis, and acquired immune deficiency syndrome were negative. Exclusion criteria were as follows: (a) acute inflammation or infection; (b) acute or chronic liver, kidney, lung, brain, heart, or other systemic diseases; (c) drug treatment history in the past month; (d) recent irregular diet, irregular working hours, insufficient sleep, or excessive drinking. This study was approved by the ethics committee of the First Affiliated Hospital of Wenzhou Medical University.

### 2.2 Laboratory methods

The venous blood collected from each subject was anticoagulated with EDTA‐K2 and tested within 2 h. According to the principle of five blood cell classification, Sysmex XE‐2100 automatic blood cell analyzer and related reagents (Sysmex) were used to determine the test items: neutrophil count, lymphocyte count, monocyte count, and platelet count in strict accordance with the instructions. NLR, LMR, and PLR are calculated as follows: NLR = neutral count/lymphocyte count,^25^ LMR = lymphocyte count/monocyte count,^26^ and PLR = platelet count/lymphocyte count.^27^ Use special reagents and standard methods. Two levels of control materials (e‐CHECK (XE)) were used daily quality control. During the whole study period, internal quality control (IQC) was conducted by Westgard multi rule quality control method. The total coefficient of variation (CV) of the two control materials was less than 5%.

### 2.3 Statistical analysis

Kolmogorov‐Smirnov test was used to test the normality of the data; the normal distribution measurement data were expressed as X±s, and the comparison between the two groups was conducted by t test; the non‐normal distribution measurement data was expressed as the median (p25‐p75), and the comparison between the two groups was conducted by Mann‐Whitney U test, and the comparison among the multiple groups was conducted by Kruskal‐Wallis H test; reference interval was established from nonparametric 95% percentile according to CLSI C28‐A3.^13^ Statistical analysis was conducted by using IBM SPSS statistics version 20.0 software and MedCalc 19.1 software. *p* < .05 was statistically significant.

## 3 RESULTS

**FIGURE 2:**
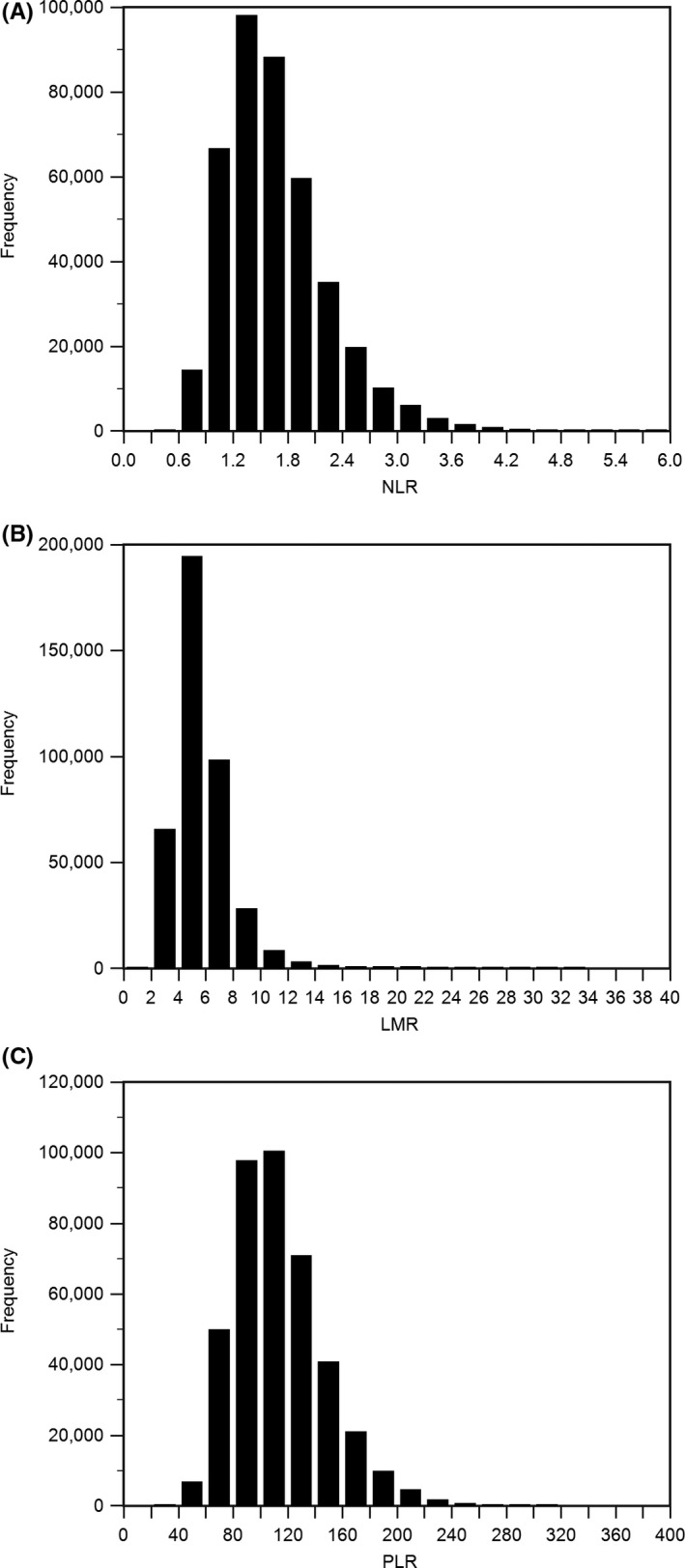
Distribution histogram of three indicators. A, Kolmogorov‐Smirnov test showed that NLR data showed skewed distribution. B, Kolmogorov‐Smirnov test showed that LMR data showed skewed distribution. C, Kolmogorov‐Smirnov test showed that PLR data showed skewed distribution

SPSS statistical results showed that among the 404,272 subjects included in the study, 205,592 were male, accounting for 50.85%; 198,680 were female, accounting for 49.15%; and the ratio of male and female was 1.03. Kolmogorov‐Smirnov test showed that NLR, LMR, and PLR data showed skewed distribution (*p* < .05) (Figure 2). In order to explore the influence of gender on NLR, LMR, and PLR, we used Mann‐Whitney U test to compare NLR, LMR, and PLR between different genders. The results showed that NLR in male (*p* < .001) was significantly higher than that in female; LMR in male (*p* < .001) and PLR in male (*p* < .001) were significantly lower than in women (Table 1).

**TABLE 1:** Sex‐dependent reference values for three indicators

Gender	Number	Median (P25‐P75)	RIs	*Z*‐value	*p*‐value
Male (NLR)	205,592	1.550 (1.252–1.933)	0–2.696	−22.093	<.001
Female (NLR)	198,680	1.587 (1.272–2.000)	0–2.805		
Male (LMR)	205,592	5.14 (4.22–6.26)	0–9.00	−71.132	<.001
Female (LMR)	198,680	5.50 (4.49–6.88)	0–10.00		
Male (PLR)	205,592	102.00 (84.70–123.24)	0–162.84	−141.920	<.001
Female (PLR)	198,680	116.57 (96.28–141.05)	0–185.52		

Abbreviations: IQR, interquartile range; RI, reference interval.

In order to explore the influence of age on NLR, LMR, and PLR, we used Kruskal‐Wallis H test to compare the NLR, LMR, and PLR of male and female on the basis of Mann‐Whitney U test results. In the male group, there were significant differences in NLR (*p* < .001), LMR(*p* < .001), and PLR(*p* < .001) in different age groups. In the female group, there were also significant differences in NLR (*p* < .001), LMR (*p* < .001), and PLR (*p* < .001) in different age groups (Tables 2, 3, 4).

**TABLE 2:** Age‐dependent and sex‐dependent reference values for NLR

Age	Number	Median (P25‐P75)	RIs	*H*‐value	*p*‐value
18–39 (male)	79,180	1.50 (1.21–1.85)	0–2.55	3245.610	<.001
40–49 (male)	60,350	1.55 (1.25–1.91)	0–2.64		
50–59 (male)	38,825	1.59 (1.28–2.00)	0–2.76		
60–69 (male)	18,404	1.67 (1.33–2.12)	0–2.99		
70–79 (male)	6791	1.74 (1.38–2.22)	0–3.17		
>80 (male)	2041	1.78 (1.39–2.26)	0–3.28		
18–39 (female)	85,970	1.59 (1.28–2.00)	0–2.80	3232.729	<.001
40–49 (female)	53,623	1.68 (1.35–2.09)	0–2.90		
50–59 (female)	36,244	1.48 (1.19–1.86)	0–2.65		
60–69 (female)	16,193	1.51 (1.21–1.90)	0–2.73		
70–79 (female)	5372	1.60 (1.25–2.04)	0–2.88		
>80 (female)	1278	1.76 (1.38–2.21)	0–3.39		

Abbreviations: IQR, interquartile range; RI, reference interval.

**TABLE 3:** Age‐dependent and sex‐dependent reference values for LMR

Age	Number	Median (P25‐P75)	RIs	*H*‐value	*p*‐value
18–39 (male)	79,180	5.29 (4.40–6.47)	0–9.48	3839.148	<.001
40–49 (male)	60,350	5.20 (4.29–6.33)	0–9.00		
50–59 (male)	38,825	5.00 (4.13–6.11)	0–8.67		
60–69 (male)	18,404	4.77 (3.90–5.82)	0–8.21		
70–79 (male)	6791	4.56 (3.69–5.67)	0–8.33		
>80 (male)	2041	4.37 (3.55–5.46)	0–7.67		
18–39 (female)	85,970	5.50 (4.50–6.87)	0–10.04	3054.759	<.001
40–49 (female)	53,623	5.25 (4.25–6.50)	0–9.50		
50–59 (female)	36,244	5.89 (4.75–7.31)	0–10.37		
60–69 (female)	16,193	5.76 (4.72–7.17)	0–10.17		
70–79 (female)	5372	5.40 (4.35–6.73)	0–9.67		
>80 (female)	1278	5.05 (4.00–6.45)	0–8.78		

Abbreviations: IQR, interquartile range; RI, reference interval.

**TABLE 4:** Age‐dependent and sex‐dependent reference values for PLR

Age	Number	Median (P25‐P75)	RIs	*H* ‐value	*p*‐value
18–39 (male)	79,180	101.30 (84.44–121.61)	0–159.55	130.254	<.001
40–49 (male)	60,350	102.33 (85.11–123.18)	0–162.43		
50–59 (male)	38,825	102.91 (85.29–124.83)	0–164.49		
60–69 (male)	18,404	103.01 (84.44–125.98)	0–170.65		
70–79 (male)	6791	100.63 (81.69–125.36)	0–169.91		
>80 (male)	2041	102.50 (81.08–127.83)	0–177.16		
18–39 (female)	85,970	115.43 (95.98–138.85)	0–181.43	3791.161	<.001
40–49 (female)	53,623	123.68 (102.22–149.32)	0–196.15		
50–59 (female)	36,244	114.09 (94.38–138.25)	0–183.47		
60–69 (female)	16,193	109.05 (90.00–132.66)	0–175.00		
70–79 (female)	5372	106.07 (86.00–130.00)	0–173.25		
>80 (female)	1278	109.96 (88.50–138.75)	0–190.46		

Abbreviations: IQR, interquartile range; RI, reference interval.

Further between‐group comparisons by Mann‐Whitney U test showed statistically significant differences between most age partitions. In the male group for NLR, the average NLR of 18–39 group was significantly lower than 40–49 group (*Z* = −19.639, *p* < .001), 50–59 group (*Z* = −31.012, *p* < .001), 60–69 group (*Z* = −41.339, *p* < .001), 70–79 group (*Z* = −34.845, *p* < .001), and >80 group (*Z* = −21.320, *p* < .001). In the female group for NLR, the average NLR of 50–59 group was significantly lower than 18–39 group (*Z* = −31.287, *p* < .001), 40–49 group (*Z* = −52.664, *p* < .001), 60–69 group (*Z* = −5.378, *p* < .001), 70–79 group (*Z* = −13.665, *p* < .001), and >80 group (*Z* = −15.770, *p* < .001). In the male group for LMR, the average LMR of >80 group was significantly lower than 18–39 group (*Z* = −26.335, *p* < .001), 40–49 group (*Z* = −23.237, *p* < .001), 50–59 group (*Z* = −18.182, *p* < .001), 60–69 group (*Z* = −10.766, *p* < .001), and 70–79 group (*Z* = −4.619, *p* < .001). In the female group for LMR, the average LMR of >80 group was significantly lower than 18–39 group (*Z* = −9.638, *p* < .001), 40–49 group (*Z* = −3.927, *p* < .001), 50–59 group (*Z* = −15.428, *p* < .001), 60–69 group (*Z* = −13.880, *p* < .001), and 70–79 group (*Z* = −6.517, *p* < .001). In the male group for PLR, the average PLR of 70–79 group was lower than 18–39 group (*Z* = −0.852, *p* = .394), 40–49 group (*Z* = −3.619, *p* < .001), 50–59 group (*Z* = −4.872, *p* < .001), 60–69 group (*Z* = −4.599, *p* < .001), and >80 group (*Z* = −0.817, *p* = .414). In the female group for PLR, the average PLR of 70–79 group was significantly lower than 18–39 group (*Z* = −20.679, *p* < .001), 40–49 group (*Z* = −35.151, *p* < .001), 50–59 group (*Z* = −17.301, *p* < .001), 60–69 group (*Z* = −6.816, *p* < .001), and >80 group (*Z* = −4.394, *p* < .001).

**FIGURE 3:**
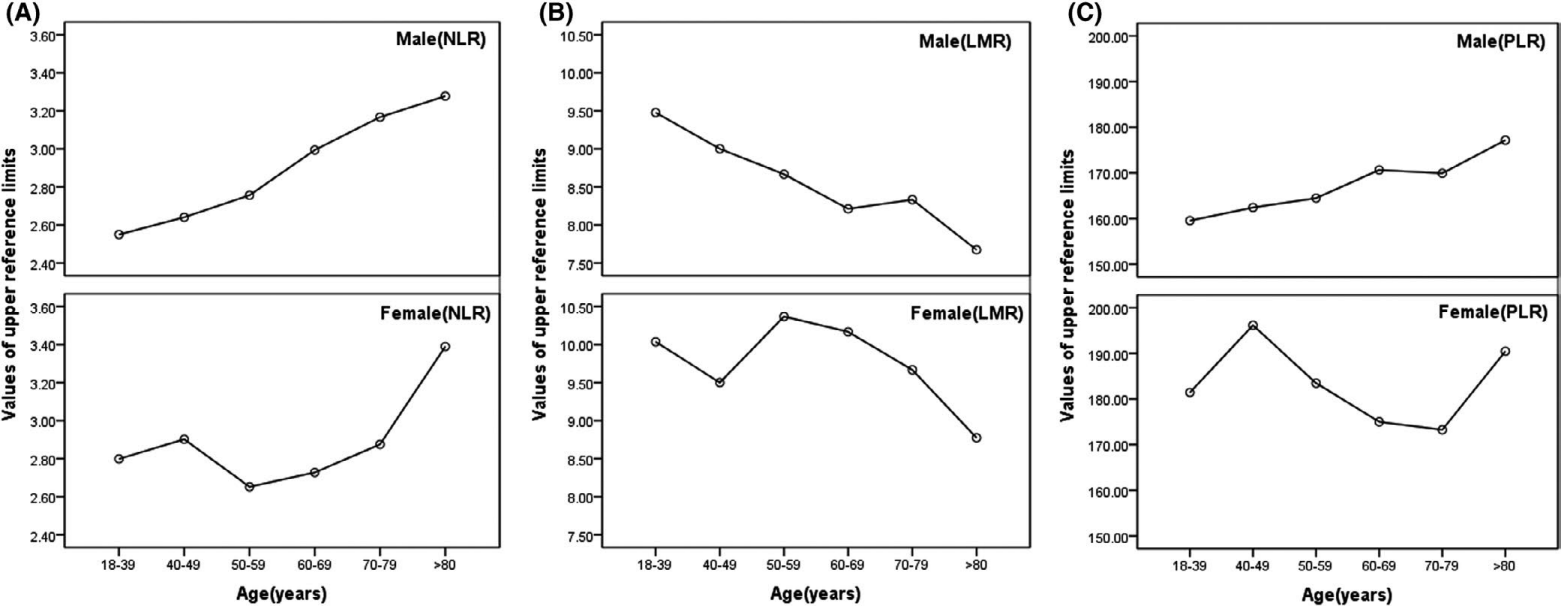
Values of upper reference limits for three indicators in different age partitions. A, The reference upper limit of NLR increased with age in male population. In female population, the reference upper limit of NLR in 50–59 group showed a trough and the reference upper limit of NLR in >80 group showed peak. B, The reference upper limit of LMR decreases with age in male population. In female population, the reference upper limit of LMR in >80 group showed a trough and the reference upper limit of LMR in 50–59 group showed peak. C, The reference upper limit of PLR increased with age in male population. In female population, the reference upper limit of PLR in 70–79 group showed a trough and the reference upper limit of PLR in 40–49 group showed peak

According to the nonparametric method recommended by CLSI CLSI C28‐A3, we established NLR, LMR, and PLR reference intervals for different genders and ages. The line chart based on age showed that the reference upper limit of NLR and PLR increased with age and the reference upper limit of LMR decreases with age in male population. In female population, the reference upper limit of NLR in 50–59 group, LMR in >80 group, and PLR in 70–79 group showed a trough; the reference upper limit of NLR in >80 group, LMR in 50–59 group, and PLR in 40–49 group showed peak(Figure 3).

## 4 DISCUSSION

In today's global pandemic of COVID‐19, NLR, LMR, and PLR, as simple and easy to measure systemic inflammatory markers, have attracted more and more attention and have been widely used in the world.^28^, ^29^

However, the results of NLR, LMR, and PLR without appropriate RI in clinical application are not valuable. Meng x^30^ only analyzed the clinical data of different disease groups in the study of PLR, but did not consider the reference range of PLR, which led to the inability to know whether the clinical data exceeded the normal reference range. Many retrospective studies have proposed "high‐risk" cutoff levels of NLR from Kaplan‐Meier curves and multivariate Cox regression analysis, but gender and age factors have not been considered. Therefore, we have established the appropriate reference interval according to gender and age, which can provide important basis for the diagnosis, progress, and prognosis of clinical diseases.

NLR, LMR, and PLR are important inflammatory markers. Different studies have shown that the NLR cutoff values for populations in western countries range from 2.5 to 5, higher than those in Asia or Africa.^31^, ^32^ Another study shows that the average NLR of Americans except non‐Hispanic black patients is higher than 2.^33^ This study shows that the average NLR of Chinese men and women is 1.550 and 1.587, respectively, which is lower than that of the United States or other western countries. It is consistent with previous studies. The reason may be related to the different natural environment, cultural environment, and eating habits in different regions. The reference upper limit of male NLR and PLR increased with age, and the reference upper limit of male LMR decreased with age, which may be due to the aging of human. The reason for this result may be the decline of immunity,^34^ the thymus involvement,^35^ and changes in T‐cell subsets,^36^ and the absolute value of lymphocytes decreased slowly. The reference upper limit of NLR, LMR, and PLR in women varies with age, which is not only the reason for the decrease of absolute value of lymphocyte representing immunity, but also related to the exuberance of sex hormone represented by estrogen and progesterone in reproductive period and the significant decrease of sex hormone in menopause. These sex hormones can increase the aggregation of neutrophils and monocytes, promote the formation of megakaryocyte polyploidy, and the formation and release of PLT precursors.^37^, ^38^

Although this study has established the reference range of NLR, LMR, and PLR based on the big data of 404,272 Chinese adults, the limitations of this study are worth emphasizing. First of all, this study is limited to the population in East China, which may make the results unrepresentative and impossible to be directly applied to subjects in other regions or clinical laboratories. In the future, it is necessary to conduct research in multiple regions and centers. Second, because this study only analyzes the data of healthy adults (>18 years old), there is no data research on adolescents, preschool children, and other special groups such as pregnant women. Third, this study also lacks more abundant data to eliminate the influence factors of these indicators, such as body mass index (BMI). In the future, we will conduct further research and collect more data.

In summary, we have established the reference range of NLR, LMR, and PLR for Chinese healthy adults of different genders and ages through big data. This will help to increase the rationality of relevant experimental research design and also help to better regulate the application of NLR, LMR, and PLR in clinical practice.

## Data Availability

All data included in this study are available upon request by contact with the corresponding author.
